# Phenotyping individuals with newly-diagnosed type 2 diabetes at risk for all-cause mortality: a single centre observational, prospective study

**DOI:** 10.1186/s13098-020-00555-x

**Published:** 2020-05-25

**Authors:** Edoardo Biancalana, Federico Parolini, Alessandro Mengozzi, Anna Solini

**Affiliations:** 1 Department of Clinical and Experimental Medicine, University of Pisa, Pisa, Italy; 2 Department of Surgical, Medical, Molecular and Critical Area Pathology, University of Pisa, Via Roma 67, 56126 Pisa, Italy

**Keywords:** Newly-diagnosed type 2 diabetes, All-cause mortality, Phenotype, Metformin, Renal function

## Abstract

**Background:**

Type 2 diabetes (T2D) shows a high mortality rate, dependent on disease duration, comorbidities and glucose control over time. Data on patients with short disease duration are scanty.

**Methods:**

We prospectively followed a cohort of newly-diagnosed T2D patients referring to a single diabetes centre, treated according to the international guidelines and checked every 6–12 months. All-cause mortality and major cardiovascular (CV) events were registered.

**Results:**

289 patients out of 3019 consecutive first attendances matched inclusion criteria and were included in the observation. Mean follow-up was 51.2 months. At 31 December 2018, 253 patients were alive and 36 deceased. At baseline, deceased individuals were older, with lower eGFR and lower uric acid, higher prevalence of atrial fibrillation. During the follow-up, 18 non-fatal CV events were adjudicated; patients with incident CV disease (CVD) differed at baseline for sex, previous history of CVD and retinopathy, higher use of secretagogues and lower use of metformin. At multivariate analysis, age and previous CVD were the only independent determinants of all-cause mortality and incident CVD, respectively. In deceased individuals, eGFR slope was markedly unstable and ΔeGFR at the end of the follow-up was higher (p < 0.001), and predicted mortality.

**Conclusion:**

Newly-diagnosed T2D patients followed according to the best clinical practice show a mortality rate similar to that reported in more complicated patients with longer disease duration; none of the clinical and biochemical variables commonly measured at baseline can predict mortality or incident CVD; early metformin use seems to be associated with no risk of prevalent or incident retinopathy.

## Background

Type 2 diabetes (T2D) is a complex disease whose prevalence shows an alarming increasing trend; in these patients the rate of all-cause and cardiovascular (CV) mortality is several folds higher than in the general population [1–3]. Various determinants of such increased risk profile can be identified; beside the presence of comorbidities like obesity and hypertension, and the degree of glucose control and of the other risk factors [4, 5], a relevant role is played by the disease duration [6–8].

The all-cause and CV risk profile in newly-diagnosed T2D is, so far, insufficiently characterized. Even though in T2D the diagnosis does not correspond to the true onset of the disease, it could be conceivable to hypothesize that, initially, the all-cause and CV mortality risk would not differ so much in comparison with non-diabetic individuals. Matter of fact that some studies have shown as in the pre-diabetes states, like impaired glucose tolerance or impaired fasting glucose, or in people with new-onset T2D, the mortality risk is superimposable, and even higher, than that observed in people with overt and long-term T2D [9–12], reinforcing the importance of an early, multifactorial intervention aimed at reducing the mortality risk in these individuals. In this view, observational studies performed in a real-world context might provide interesting and useful information on the clinical and biochemical parameters marking deaths or major CV events occurring over a relatively short time, including the role of a very early vs a late start of the pharmacologic treatment [13], allowing an early identification of high-risk patients in which appropriate and timely interventions might help to reduce the risk of premature mortality. The aim of this study was to explore whether, among a cohort of patients with new-onset T2D referring to a diabetes clinic and treated according to the international guidelines for all CV risk factors, a clinical phenotype able to predict the risk of early death or occurrence of CV events over a relatively short follow-up (FU) could be identified.

### Subjects and methods

This observational, prospective, single centre study enrolled all the patients referring for the first time to the outpatient diabetes clinic in the department of Internal Medicine between January 2008 and June 2015 and matching the inclusion criteria, *i.e.* age ≥ 30 years and personal history of known T2D lasting not more than 6 months. Main exclusion criteria were type 1 diabetes and other specific types of diabetes; acute complications of diabetes, such as diabetic ketoacidosis or lactic acidosis; autoimmune diseases; severe mental illness. Diagnosis was confirmed on the basis of the OGTT or HbA1c ≥ 6.5% plus fasting blood glucose ≥ 126 mg/dl. According to such criteria, 289 patients out of 3019 (9.6%) were identified and included in the prospective observation. The study protocol was approved by the Ethics Committee of the University of Pisa, and an informed consent was obtained by the participants.

Patients underwent a complete clinical examination, annotating current glucose, blood pressure (BP) and lipid lowering therapy, with indication of the class of drug. BMI was calculated, BP and vital parameters were registered, and blood samples were collected for routine analyses. Complete blood count, glucose, HbA1c, transaminases, uric acid, were determined by standard techniques. Plasma cholesterol and triglycerides concentrations were measured by standard enzymatic assays with commercially available kits. HDL‐cholesterol was measured after precipitation of ApoB containing lipoproteins, allowing for the estimate of LDL‐C by the Friedewald formula. Serum creatinine was measured by the isotope dilution mass spectrometry (IDMS) traceable method; glomerular filtration rate was estimated (eGFR) using the CKD‐EPI equation. Within 1 week from the basal evaluation, patients underwent also an ophthalmoscopic exam (to test the presence of background or advanced retinopathy).

Previous major acute CV events, including myocardial infarction, stroke, foot ulcer/gangrene/amputation and coronary, carotid and lower limb revascularisation, were adjudicated based on hospital discharge records. Heart failure was defined as left ventricular ejection fraction ≤ 45% [14], and/or previous hospitalization for heart failure. Atrial fibrillation was adjudicated only when permanent or recurring.

*Follow up* At the end of the baseline visit, patients were treated according to the good clinical practice recommended by the international guidelines, and followed a 6-month or an yearly calendar of follow-up visits, until death or until 31 December 2018. All-cause mortality was assessed by checking the vital status of study participants on 31 December 2018; for this purpose, we interrogated the Italian Health Card database (http://sistemats1.sanita.finanze.it/wps/portal/), which provides updated information on all current Italian residents. Incident major acute CV events were registered on the basis of clinical records every year; retinopathy onset was assessed by fundoscopy on a yearly basis. Seven patients were excluded from such analyses due to missing information. HbA1c and eGFR slopes were built up to the end of the follow up.

*Statistics* Data are expressed as mean (SD) or median (interquartile range) for continuous variables, and number of cases (percentage) for categorical variables. Continuous variables were compared using one-way ANOVA or the nonparametric Kruskal–Wallis test. The χ^2^ test was applied to categorical variables. Annual mortality rate (‰) was calculated as the number of deaths/(population size over the follow-up period*mean duration of follow-up expressed in years)*10^4^. To assess correlations, we used univariable and multivariable logistic regression models, adjusting for confounders (sex, age, BMI, blood pressure, glycaemia, HbA1c, uric acid), reporting results as Odds Ratio [95% C.I.]. Statistical tests were conducted using a two-sided α-level of 0.05.

## Results

Baseline clinical characteristics of the newly-diagnosed participants are shown in Table 1. Patients were adequately distributed for sex. Smoking habits were highly prevailing in males. Women were more obese and showed a worse lipid profile; however, LDL cholesterol was far from being at target (< 100 mg/dl in primary prevention according to international guidelines) in both sexes. 25.3% of the study group showed a scarce metabolic control (HbA1c > 8%), with no sex difference in fasting blood glucose or HbA1c. Renal function was similar in men and women; 13.2% of patients were already in a CKD stage ≥ 3 according to eGFR. Retinopathy was present only in seven patients, all males.

**Table 1:** Clinical and biochemical characteristics of the study cohort at baseline

	All 289	Men 158 (54.7%)	Women 131 (45.3%)	p value
Age (years)	63.3 ± 11.0	62.7 ± 12.0	64.0 ± 11.0	ns
Ethnic group (caucasian/black/Asian; %)	95/2/3	94/3/3	97/1/2	ns
High school education or above (n; %)	101; 34.9	53; 33.5	48; 36.6	ns
BMI (kg/m^2^)	30.5 ± 6.0	29.5 ± 5.3	31.7 ± 6.5	0.0026
Smoking habits (yes/no/ex; %)	11/51/38	15/38/47	5/69/26	< 0.001
SBP (mmHg)	141.0 ± 19.7	140.0 ± 19.4	142.2 ± 20.3	ns
DBP (mmHg)	83.0 ± 11.0	83.1 ± 11.0	83.0 ± 11.1	ns
Fasting glucose (mg/dL)	158 ± 57	158 ± 60	157 ± 54	ns
Hb1Ac (%)	7.6 ± 1.7	7.6 ± 1.6	7.7 ± 1.8	ns
Patients with HbA1c > 8% (n; %)	73; 25.3	43; 27.2	30; 22.9	ns
Total cholesterol (mg/dL)	202 ± 43	192 ± 41	214 ± 44	< 0.0001
HDL-cholesterol (mg/dL)	48 ± 16	43 ± 11	55 ± 18	< 0.0001
LDL-cholesterol (mg/dL)	122 ± 42	114 ± 42	131 ± 41	0.0022
Triglycerides (mg/dL)	169 ± 106	179 ± 115	157 ± 92	ns
ALT (IU/L)	32 ± 22	34 ± 25	30 ± 19	ns
AST (IU/L)	24 ± 13	26 ± 13	23 ± 12	ns
γGT (IU/L)	50 ± 59	57 ± 73	43 ± 36	ns
Serum creatinine (mg/dL)	0.95 ± 0.41	1.1 ± 0.45	0.77 ± 0.16	< 0.0001
eGFR CKD-EPI (mL/min/1.73 m^2^)	79.2 ± 21.0	77.3 ± 23.0	81.5 ± 18.0	ns
eGFR < 60 mL/min/1.73 m^2^ (n; %)	38; 13.2	23; 14.6	15; 11.5	ns
Uric acid (mg/dL)	6.0 ± 1.8	6.3 ± 1.8	5.6 ± 1.5	ns
Diabetic retinopathy (n; %)	7; 2.4	7; 4.4	0; 0	0.022
Previous CV disease (n; %)	44; 15.2	37; 23.4	7; 5.3	< 0.0001
Heart failure (n; %)	19; 6.6	12; 7.6	7; 5.3	ns
Atrial fibrillation (n; %)	23; 8.0	15; 9.5	8; 6.1	ns
Any cancer (n; %)	50; 17.5	23; 14.6	27; 21.1	ns

On the basis of clinical records, a previous CV event was confirmed in 15% of the patients, with a significant prevalence in the male sex. Prevalence of heart failure and atrial fibrillation was 7% and 8%, respectively.

Table 2 shows the initial distribution of the pharmacologic treatments. At baseline visit, 54% of patients did not assume any treatment for diabetes. Metformin (mean dose 1000 mg/die) was already on board in one-third of the patients, while 17.3% of them received secretagogues, alone or in combination with metformin; in these latter, HbA1c was significantly higher vs those not receiving the drugs (8.1 ± 2.0 vs 7.3 ± 1.3%, p = 0.012). Sixty-two percent of the patients received anti-hypertensive treatment; despite the insufficient control of lipid levels, only 30% of the patients were on statin treatment.

**Table 2:** Pharmacologic treatment of the study cohort at baseline

	All 289	Men 158 (54.7%)	Women 131 (45.3%)	p value
No treatment (n; %)	155; 53.6	81; 51.3	74; 56.5	ns
Metformin (n; %)	84; 29.1	48; 30.4	36; 27.5	ns
Secretagogues (± metformin; n; %)	50; 17.3	29; 18.4	21; 16.0	ns
Anti-hypertensive treatment (n; %)	179; 61.9	103; 65.2	76; 58.0	ns
Statins (n; %)	88; 30.4	50; 31.6	38; 29.0	ns

*Follow up* Mean duration of the follow up was 51.2 months (median: 49 months); during such observation, we registered 36 deaths (12.5% of the study population). The mortality rate in this cohort was 29.2 ‰/year. Table 3 shows the baseline phenotype of patients alive or deceased at the index date. Patients encountering exitus within four years from the diagnosis of T2D were older, had a remarkably lower eGFR, lower uric acid levels, and a higher prevalence of atrial fibrillation. No significant differences emerged in the distribution of the treatments for T2D and comorbidities, as well as in the mean HbA1c, even though in the group of deceased patients there was a significant greater percentage of patients with impaired glycaemic control (HbA1c > 8%) at baseline. After multivariate analysis, age at diagnosis remained as the only basal predictor of all-cause mortality.

**Table 3:** Baseline phenotype and treatments of patients deceased or alive over the follow up

	Deceased 36 (12.8%)	Alive 253 (87.2%)	p value
Age (years)	72.8 ± 9.0	61.9 ± 11.3	< 0.0001
Sex (n; %)	F 14 (38.9%) M 22 (61.1%)	F 117 (46.2%) M 136 (53.8%)	ns
BMI (kg/m^2^)	30.0 ± 5.8	30.6 ± 6.0	ns
SBP (mmHg)	145.6 ± 19.8	140.3 ± 19.7	ns
DBP (mmHg)	84.8 ± 11.3	82.8 ± 11.0	ns
Fasting glucose (mg/dL)	156 ± 50	158 ± 59	ns
Hb1Ac (%)	7.9 ± 1.3	7.6 ± 1.8	ns
Patients with HbA1c > 8% (n; %)	15; 45.5	58; 25.5	0.0174
Total cholesterol (mg/dL)	196 ± 40	203 ± 44	ns
HDL-cholesterol (mg/dL)	51 ± 14	48 ± 16	ns
LDL-cholesterol (mg/dL)	122 ± 53	122 ± 41	ns
Triglycerides (mg/dL)	136 ± 75	173 ± 108	ns
ALT (IU/L)	25 ± 12	33 ± 23	ns
AST (IU/L)	21 ± 7	25 ± 13	ns
γGT (IU/L)	44 ± 28	51 ± 62	ns
Serum creatinine (mg/dL)	1.06 ± 0.6	0.93 ± 0.37	ns
eGFR CKD-EPI (mL/min/1.73 m^2^)	70.6 ± 19.2	81.0 ± 20.0	0.0256
eGFR < 60 mL/min/1.73 m^2^ (n; %)	8; 22.2	30; 11.9	ns
Uric acid (mg/dL)	4.9 ± 1.1	6.2 ± 0.2	0.0291
Diabetic retinopathy (n; %)	1; 2.8	6; 2.4	ns
Previous CV disease (n; %)	6; 16.7	38; 15.0	ns
Heart failure (n; %)	4; 11.1	15; 5.9	ns
Atrial fibrillation (n; %)	6; 16.7	17; 6.7	0.0067
Any cancer (n; %)	10; 27.8	40; 15.8	ns
No treatment (n; %)	16; 44.4	139; 54.9	ns
Metformin (n; %)	12; 33.3	72; 28.5	ns
Secretagogues (n; %)	8; 22.2	42; 16.6	ns
Anti-hypertensive treatment (n; %)	23; 63.9	156; 61.6	ns
Statins (n; %)	14; 38.9	74; 29.2	ns

**Fig. 1:**
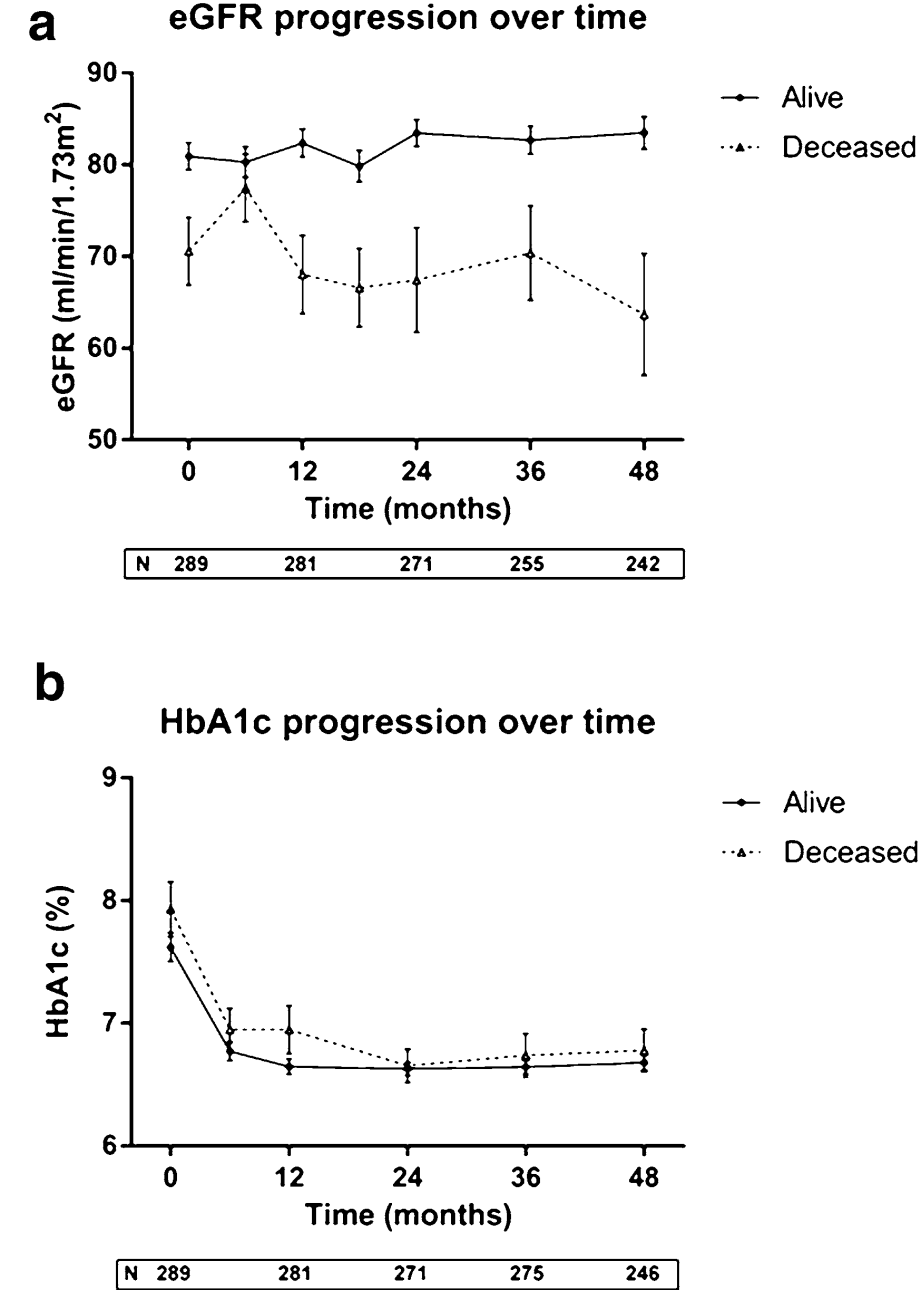
Glomerular function (**a**) and HbA1c (**b**) over time in relation to mortality

*Role of the renal phenotype* Glomerular function strongly influences the prognosis in T2D; even though eGFR was not an independent predictor of all cause-mortality in this cohort of patients with short T2D duration, we explored its behaviour over time by building up the slopes on the basis of serum creatinine measured every six months, and relating them to all-cause mortality. Data are shown in Fig. 1a; interestingly, ΔeGFR at 52 months was significantly higher in deceased individuals (− 6.9 ± 3.8 vs +2.5 ± 0.1 mL/min/1.73 m^2^, p < 0.001); moreover, eGFR slope in these subjects was markedly unstable, with ample oscillations over the observation period (SD: 6.3 [4.8–12.3] vs 5.6 [3.5–8.5] mL/min/1.73 m^2^); the slope of alive individuals remained virtually stable. In the multivariate analysis, ΔeGFR emerged as significant determinant of mortality (OR 1.14 [1.01–1.43], p = 0.0453) also when adjusted for confounders (sex, age, BMI, blood pressure, glycaemia, HbA1c, uric acid). We also checked the impact of metabolic control over time, but the mean HbA1c value registered during the whole follow up (mean 6.3 determinations/patient) did not differently marked deceased vs surviving individuals (Fig. 1b).

*Phenotype and incident CV events* Table 4 compares the baseline phenotype of people developing or not an acute CV event over the follow up; seven missed patients were excluded from this analysis. We registered 18 new CV events; 50% of them were first episodes, and 50% recurrent events. Only 3 events occurred in patients deceased during the follow-up, while the remaining were surely non-fatal events. Clinical characteristics marking such events were the male sex, the previous personal history of CV disease and retinopathy and, interestingly, a relative delay in the time of starting the antidiabetic treatment; in detail, macrovascular events occurred in a significantly higher percentage in patients not pharmacologically treated since the very beginning of the disease, or in those receiving sulphonylureas. As expected, multivariate analysis identified previous CV events (OR 21.51 [1.37–338], p = 0.0097) as the only independent predictor of future CV events in such study cohort; predictive elements for incident CVD in people with no previous personal history were undetectable.

**Table 4:** Baseline phenotype of patients developing or not developing acute CV events over the follow up **(**seven missing patients excluded from the analysis)

	New CV events 18 (6.4%)	No new CV events 264 (93.6%)	p value
Age (years)	66.6 ± 8.0	63.0 ± 11.8	ns
Sex (n; %)	F 3; 16.6% M 15; 83.2%	F 122; 46.2% M 142; 53.8%	0.0146
BMI (kg/m^2^)	28.7 ± 5.0	30.5 ± 5.9	ns
SBP (mmHg)	142.7 ± 21.2	140.5 ± 19.7	ns
DBP (mmHg)	81.4 ± 10.4	83.0 ± 11.0	ns
Fasting glucose (mg/dL)	138 ± 51	159 ± 58	ns
Hb1Ac (%)	7.3 ± 1.0	7.7 ± 1.8	ns
Total cholesterol (mg/dL)	185 ± 29	203 ± 43	ns
HDL-cholesterol (mg/dL)	43 ± 11	48 ± 14	ns
LDL-cholesterol (mg/dL)	114 ± 27	123 ± 43	ns
Triglycerides (mg/dL)	140 ± 49	170 ± 108	ns
ALT (IU/L)	25 ± 9	32 ± 23	ns
AST (IU/L)	23 ± 4	25 ± 13	ns
γGT (IU/L)	41 ± 25	50 ± 60	ns
Serum creatinine (mg/dL)	1.07 ± 0.3	0.94 ± 0.42	ns
eGFR CKD-EPI (mL/min/1.73 m^2^)	71.8 ± 17.3	80.0 ± 21.2	ns
eGFR < 60 mL/min/1.73 m^2^ (n; %)	3; 16.7	34; 12.9	ns
Uric acid (mg/dL)	6.6 ± 1.2	5.9 ± 1.8	ns
Previous CV disease (n; %)	9; 50.0	33; 12.5	< 0.0001
Heart failure (n; %)	3; 16.7	15; 5.7	ns
Atrial fibrillation (n; %)	1; 5.6	22; 8.3	ns
Any cancer (n; %)	5; 27.8	45; 17.0	ns
Diabetic retinopathy (n; %)	3; 16.7	4; 1.5	< 0.0002
No treatment (n; %)	8; 44.4	143; 54.2	0.0400
Metformin (n; %)	3; 16.7	79; 29.9	
Secretagogues (n; %)	7; 38.9	42; 15.9	
Anti-hypertensive treatment (n; %)	13; 72.2	160; 60.1	ns
Statins (n; %)	7; 41.1%	76; 29.0%	ns

Table 5 shows the prevalence of macrovascular complications and retinopathy at baseline and at the end of the observation period, according to baseline treatment for T2D. Early use of metformin was coupled, even in this small cohort, to less incidence of CV events over the follow up. Of note, no retinopathy was present at diagnosis, neither developed at follow up (n = 13), in patients starting an early treatment with metformin.

**Table 5:** Patients with CVD, retinopathy and CKD stage ≥ 3 at baseline and at the end of the follow up according to treatment of T2D at baseline (seven missing patients excluded from the analysis)

	All	No treatment 148 (53.6%)	Metformin 84 (29.1%)	Secretagogues 50 (17.3%)	p value
Baseline CV disease (n; %)	44	26; 17.6	9; 10.7	9; 18.0	ns
Follow up CV disease (n; %)	18	8; 5.4	3; 3.6	7; 14.0	0.04
Baseline any retinopathy (n; %)	7	3; 2.0	0; 0	4; 8.0	0.015
Follow up any retinopathy (n; %)	13	7; 4.7	0; 0	6; 12.0	0.007
Baseline CKD stage ≥ 3	38	21; 14.2	9; 10.7	8; 16.0	ns
Follow up CKD stage ≥ 3	17	9; 6.1	2; 2.4	6; 12.0	ns

## Discussion

The main results of our single-centre, real-life prospective observation can be summarized as follows: (*i*) in a cohort of newly-diagnosed T2D individuals, the mortality rate in the first years after the diagnosis does not differ from that reported in large cohorts of patients with longer disease duration; (*ii*) a quite large panel of clinical and biochemical variables at baseline does not allow to predict mortality or incident CVD; (*iii*) early metformin use seems to be associated with no risk of prevalent or incident retinopathy.

Real-life observations performed in large cohorts of T2D individuals indicate a variable mortality rate, higher in the Western countries [15–17] and lower in Arab countries [18], and declining over the last decade [19]; diabetes duration is, indeed, one of the main determinants of such risk [20, 21]. In our small sample we show that, despite the intensive treatment of all risk factors and comorbidities, the mortality rate of the patients over the first 5 years of known disease is superimposable to that of patients with much higher diabetes duration and prevalence of comorbidities, confirming the relative weight of prediabetes state in determining a higher CV and, of less extent, mortality risk [10, 22, 23]. Another possibility could be a short-term negative effect on mortality that follows the diagnosis of diabetes in older adults; such phenomenon was not reported in the large subset of the Kaiser Permanente Northern California with the same age of our patients and a short diabetes duration, where the mortality rate was 19.61/1000 patients/year [24]. This early, relatively high mortality risk is compelling, because implies that even a brief exposure to hyperglycaemia could elicit detrimental effects, and that macrovascular damage through atherosclerotic pathways may not be primarily responsible.

The alarming picture is made even worse by the lack of any predictive value on the short-term mortality of an ample panel of clinical and biochemical routine variables, like metabolic control and previous CVD. Age at diagnosis was, actually, the only independent predictor; its prognostic implications have been recently pointed out in a key report from the Swedish National Diabetes Registry [25], and reinforces the need for a capillary screening and an early identification of any derangement of glucose tolerance, hoping that an early treatment aimed at targeting the various risk factors would be able to reduce the mortality risk in such individuals. Moreover, in this small cohort of individuals adequately treated for concomitant risk factors, incident CV events have not been easily predictable in those without previous personal history of CVD, pointing out an urgent need for finding novel, easily-measured, reliable biomarkers.

Interestingly, in these subjects an early use of metformin seems to be associated with absence of retinopathy over the whole observation period. Recent retrospective observations in patients with longer disease duration have documented a reduced severity of retinopathy in those receiving metformin [26]. Metformin was found effective also in protecting toward other forms of eye involvement in T2D [27, 28]; it should be also pointed out as, in the Diabetes Prevention Program Outcomes Study, prolonged metformin treatment in subjects with pre-diabetes did reduce the incidence of T2D, but not of the aggregate microvascular outcome [29]. Prospective real-life data in patients with short disease duration are scanty; our observation, that need to be confirmed on larger numbers, seems to support the idea that an early use of metformin might protect the eye, likely because it does not induce hypoglycaemia, detrimental toward the retina [30], or—alternatively—by exerting endothelial protection [31].

Trend of eGFR in our study cohort deserves a comment, being the delta between basal and final value significantly higher in T2D patients encountering death over the follow-up; even more, multivariate analysis confirmed it as predictor of mortality. This supports the notion that a fast decline of glomerular filtration, even in subjects with short duration of T2D and preserved renal function, is a main factor affecting the short as well as long-term prognosis [32, 33], reinforcing the need for early therapeutic strategies of nephroprotection.

We should acknowledge several limitations of the present study: the small cohort, the lack of data on albuminuria (according to guidelines and good clinical practice rules, albuminuria should be tested in T2D within the first year from the diagnosis, while our baseline data refers to the first observations performed in our diabetes centre, and almost none had such determination at that time).

## Conclusions

In conclusion, our findings may offer considerations for the management and prevention of CV events and mortality in T2D. The evidence of a high mortality risk in newly diagnosed patients, and the relatively low impact of screening for diabetes on mortality [34] emphasizes the need to be more effective in early treatments. These findings also highlight the role of age, both for risk stratification and management, and irrespective of the duration of the disease, and reinforce the need for interventions aimed at slowing the decline of renal function immediately after the diagnosis.

## Data Availability

The dataset analyzed during the current study is available from the corresponding author upon reasonable request.

## Abbreviations

CKD: Chronic kidney disease
CV: Cardiovascular
CVD: Cardiovascular disease
eGFR: Estimated glomerular filtration rate
T2D: Type 2 diabetes
